# Expression of Telomerase and Telomere Length Are Unaffected by either Age or Limb Regeneration in *Danio rerio*

**DOI:** 10.1371/journal.pone.0007688

**Published:** 2009-11-06

**Authors:** Troy C. Lund, Tiffany J. Glass, Jakub Tolar, Bruce R. Blazar

**Affiliations:** Division of Pediatric Blood and Marrow Transplantation Program, University of Minnesota, Minneapolis, Minnesota, United States of America; Texas A&M University, United States of America

## Abstract

**Background:**

The zebrafish is an increasingly popular model for studying many aspects of biology. Recently, *ztert*, the zebrafish homolog of the mammalian telomerase gene has been cloned and sequenced. In contrast to humans, it has been shown that the zebrafish maintains telomerase activity for much of its adult life and has remarkable regenerative capacity. To date, there has been no longitudinal study to assess whether this retention of telomerase activity equates to the retention of chromosome telomere length through adulthood.

**Methodology/Principal Findings:**

We have systematically analyzed individual organs of zebrafish with regard to both telomere length and telomerase activity at various time points in its adult life. Heart, gills, kidney, spleen, liver, and intestine were evaluated at 3 months, 6 months, 9 months, and 2 years of age by Southern blot analysis. We found that telomeres do not appreciably shorten throughout the lifespan of the zebrafish in any organ. In addition, there was little difference in telomere lengths between organs. Even when cells were under the highest pressure to divide after fin-clipping experiments, telomere length was unaffected. All aged (2 year old) tissues examined also expressed active amounts of telomerase activity as assessed by TRAP assay.

**Conclusions/Significance:**

In contrast to several other species including humans, the retention of lifelong telomerase and telomeres, as we have reported here, would be necessary in the zebrafish to maintain its tremendous regenerative capacity. The ongoing study of the zebrafish's ability to maintain telomerase activity may be helpful in unraveling the complexity involved in the maintenance (or lack thereof) of telomeres in other species such the mouse or human.

## Introduction

The study of telomere biology is important as it relates to the process of aging and the ability to repair tissue. In humans most of our somatic tissues lack telomerase, the enzyme responsible for maintaining the telomeric repeat elements at the ends of our chromosomes. Consequently, with each cell division telomere ends shorten, and hence, the chromosomes as well. When telomeres reach a critical length, cells undergo a cessation in division known as cellular senescence and can develop abnormal karyotypes [1], [2]. The inability to maintain the ends of our chromosomes in somatic cells contributes to the lack of true regenerative ability in human tissue as compared with other organisms such as the zebrafish. There is a small population of human cells that do retain telomerase which are tissue specific stem cells; they are responsible for repairing damaged tissues and must continually self-renew themselves and regenerate the progenitors for which they are programmed [3]. Also, many human cancer cells have adopted the ability to constitutively express telomerase which assists in their ability to remain immortal [4].

Recently, the zebrafish, *Danio rerio*, has become an increasingly popular model to examine a variety of diseases including those relating to aging, tissue repair, and regeneration [5], [6], [7], [8]. The zebrafish gene for telomerase, *ztert*, has been cloned and found to be quite conserved in sequence between humans and fish. Zebrafish have been shown express telomerase in the skeletal muscle even at 24 months of age [8].

Although recent studies have shown the zebrafish to be a potentially useful model to study cellular senescence [6], there have not been longitudinal studies evaluating telomeres or telomerase in zebrafish. We now show that zebrafish do not undergo telomere shortening when evaluated from 3 to 24 months. This is likely due, at least in part, to the fact that they express telomerase in all tissues evaluated at 24 months of age. Furthermore, fin-clipping experiments performed to analyze the stability of telomeres under a large cellular division pressure showed no decline in telomere length in regenerated tissue after successive fin-clips. This constitutive expression of telomerase and telomere retention throughout life contributes, in part, to the zebrafish's ability to regenerate tissues.

## Results

**Figure 1:**
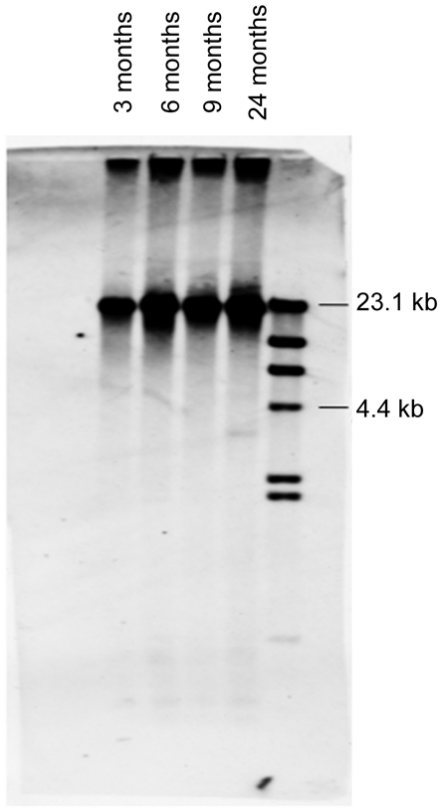
Telomeres in the zebrafish liver do not shorten with age. 1 µg of DNA prepare from liver was digested with Hinf II, Rsa I, Alu I, Hae I, and Msp then separated on a 0.5% agarose gel by pulse-field gel electophoresis, transferred to nylon, probed with a Dig-labeled telomere probe (TTAGGG)_3_, and detected via chemiluminescense. Organs from three fish were pooled for each age group.

**Figure 2:**
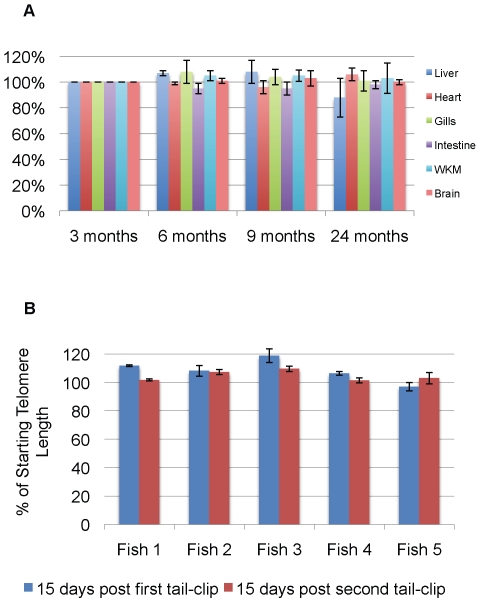
Telomeres in various zebrafish organs do not shorten with age or after successive fin-clippings. Panel A. DNA was prepared from organs (three organs per age group), restriction enzyme digested as before, and Southern blot performed as described in Figure 1. Telomere length was determined by scanning densitometry and telomere signals quantified using Telometric™ software. Data is displayed as change from the initial telomere measurement at three months of age (given as 100%). Four independent experiments were performed for each age group and telomere measurements averaged. Error bars are standard deviations. WKM denotes whole kidney marrow. Panel B. Telomere DNA analysis from individual tailfins is shown after two successive rounds of tail-clipping each about 15 days apart when the tail is fully regenerated. Data is displayed as percent change from the preclip telomeric length. Three measurements of each fin were performed and averaged. Error bars are standard deviations.

To study whether or not zebrafish had telomere shortening throughout their life and to compare various organs, DNA was extracted from different organs at various time points and telomere length was determined by Southern blotting. Figure 1 shows the average telomere length in the liver of various aged fish. Two observations can be made: (1) the telomeres are longer than one would expect in human cell types at nearly 20–25 kb, and (2) there appears to be no significant shortening with age. The graph in figure 2 shows our analysis of several tissues with regards to telomere length over time. No significant changes occurred up to 24 months of age in the brain, kidney, liver, or heart (Figure 2A).

It is known that fish can regenerate their fins and other organs quite readily [9], [10], [11]. Other teleosts such as the killifish have been shown to upregulate telomerase during fin regeneration [12]. We were curious to know if the very high cellular division required with this type of regeneration would cause telomere erosion. Figure 2B shows that even after 2 cycles of fin-clipping there is minimal telomere shortening in any of the individual zebrafish analyzed.

**Figure 3:**
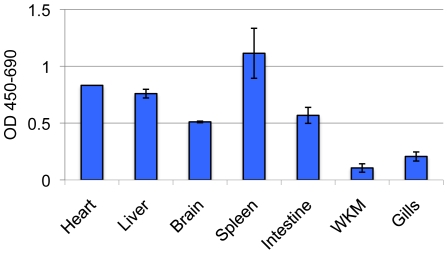
Telomerase activity is present in the organs of aged fish. Pooled organs from 3 fish per age group were dissected and flash frozen in liquid nitrogen until used. Protein was extracted using CHAPS buffer and 1 ug of total protein used in a TRAP assay. Results are shown as OD (450–690) above background (Rnase treated lysates). All samples performed in duplicate. WKM denotes whole kidney marrow.

The maintenance of telomeres requires active telomerase in most cases, and it has been described that like human embryonic tissues zebrafish embryos have active telomerase [13], [7]. In addition zebrafish telomerase activity has been seen in “adult” tissues of brain, heart, kidney, heart, gill, muscle and skin although the age was not given in a prior study [14]. Therefore, we next sought to verify that the aged fish we were analyzing also had telomerase enzyme activity. Figure 3 shows that all tissues examined by TRAP assay had some level of telomerase activity at 24 months of age. There was a range of telomerase activity in the various organs with the highest levels seen in the spleen, heart and liver, intermediate levels in the brain and intestine, and the lowest levels in the whole kidney marrow and gills. This retained constitutive activity of telomerase probably accounts for the lack of telomere shortening in the tissues of the aged fish.

## Discussion

This is the first demonstration of the retention of telomerase and telomere length as measured in various organs longitudinally in the zebrafish. Our finding of telomere retention is in contrast to the recent finding in the teleost medaka that although they retain telomerase activity in their tissues and organs, the fish show telomere shortening with age[15]. Why telomere attrition occurs in medaka but not *danio* is not clear, but may be species-specific. In support of a possible species-specific telomere attrition, there does not appear to be a strong correlation between telomerase expression and lifespan in another teleost, *Nothobranchius furzeri, which is strain specific* [16]. In comparison to other strains, its has been shown that one strain of *Nothobranchius furzeri* showed telomerase expression and activity despite aging and having a shorter lifespan. Additionally, it is also possible that the length and retention of telomeres is specific to the strain of vertebrate species that is housed and bred in a given animal facility. This is evidenced by the fact that mice of different strains can have varied telomere length measurements [17], [18].

The zebrafish in our analysis seem to have telomere regulation much like that in mice, which also have very long telomeres that do not seem to shorten significantly during life. Murine telomeres can be induced to shorten either by introduction of a chromosomal aberration (e.g. by carcinogen or radiation) or when telomerase is disabled by gene knock-out [19], [20].

The ability of the zebrafish to undergo fin and organ regeneration requires the cells performing the repair to undergo many cell divisions [9]. To prevent the inescapable telomere attrition that would accompany this, it seems logical that the zebrafish has retained constitutive telomerase activity in its organs to preserve telomere length. Our results show telomerase activity in all tissues examined, though there is somewhat lower activity in the whole kidney marrow and gills compared to the other organs. However, this degree of difference in telomerase activity between organs is not sufficient to result in substantial differences in telomere length. It is possible that the more differentiated cells of the marrow, i.e. myelomonocytes (which are neutrophil equivalents) have lower telomerase activity and bring down the total level [21]. It is also possible that an inhibitor of the assay was present in the whole kidney marrow and gills.

Haploinsufficiency for telomerase results in shortened telomeres in human disease such as dykeratosis congenita, a congenital and progressive bone marrow failure syndrome due to a genetic defect in small nucleolar riboproteins, although the absolute telomerase activity level has not been reported to date [22]. Some progress has been made to determine an absolute telomerase level in the yeast system by Mozdy *et al.* who found that telomere length is very sensitive to the amount of telomerase present, and only a twofold change in telomerase abundance can affect elongation [23]. We have found all tissues examined have long telomeres irrespective of the exact level of telomerase activity demonstrated in the various tissues of the zebrafish when analyzed at 24 months of age. The variances in telomerase activity may be due to the replicative history of the exact tissue analyzed such that tissues that have not experienced prior high levels of mitotic activity will retain longer telomeres irrespective of the exact levels of telomerase activity observed. While such an explanation may seem likely for tissues with low cell turnover such as brain, for tissues with high turnover (e.g. intestine), we favor the explanation that telomerase activity may be above a yet undetermined threshold required to maintain telomeric ends.

Others have found in killifish tail-clip experiments telomerase is up-regulated, and telomeres could even lengthen after this type of injury and regeneration [12]. Our data agrees with the finding in that telomeres do not shorten appreciably after tail-clip and subsequent tailfin regeneration. It is hypothesized that retention of telomerase activity and telomere length is related to the zebrafish's regenerative capacity, which is an ability that mammals lack.

The zebrafish has been studied as a model of aging. During aging, zebrafish have been shown to be susceptible to oxidative stress and to display biomarkers associated with cellular senescence including increased b-galactosidase activity. Genetic screening has been done in zebrafish, and mutants have been found that display early biomarkers of senescence and have shortened life spans as heterozygotes[6]. Our data indicating that the lack of shortening of telomeres with age in the zebrafish are of importance to those using the zebrafish as a model of aging, especially for those studies relating aging mechanisms in zebrafish to those in humans.

Clearly, there are a multitude of factors contributing to the aging process: telomere attrition, oxidation, post-translational protein modifications, and DNA damage. Our data leads us to the conclusion that telomere shortening probably does not play an obligatory role in teleost aging. Nonetheless, much can be learned from the fact that telomeres and telomerase are retained lifelong in the zebrafish, particularly with respect to the mechanisms involved in organ/tissue regeneration, which can be readily studied in the zebrafish.

## Materials and Methods

Zebrafish were housed under standard laboratory conditions in a core facility. Water temperature was maintained at 28 C. Ethical approval was obtained from the Institutional Animal Care and Use Committee of the University of Minnesota. The wild-type fish used were derived from zebrafish originally obtained from Segrest Farms (Gibsonton, FL) maintained in our facility.

### Telomere Length Analysis

Various organs were dissected from aged zebrafish and flash-frozen in liquid nitrogen until DNA was prepared. We pooled three organs per age group except in the tail-clip experiments in which each individual tail was prepared separately. DNA was prepared using a Gentra® Puregene® kit (Qiagen, Valencia, CA) One microgram of DNA was digested with Hinf II, Rsa I, Alu I, Hae I, and Msp; then separated on 0.5% agarose gels by pulse-field electrophoresis and transferred to positively-charged nylon membrane by vacuum in alkaline buffer. Telomere length was determined by probing with a digoxigenin (DIG) end-labeled telomere probe (TTAGGG)_3_ at 42 C for 12 hours in DIG Easy Hyb buffer (Roche, Indianapolis, IN). Membranes were washed, blocked, incubated with anti-DIG antibody (Roche), and developed by chemiluminescence (Roche). Average telomere length was determined by scanning densitometry and Telometric™ software.

### Telomerase Activity

Organ lysates were prepared (three organs per age group) in CHAPS lysis buffer and TRAP assay was performed using Telomerase PCR ELISA assay from Roche per the manufacturer's instructions using 1 µg of whole cell lysate. Results are displayed as telomerase activity above background (lysates treated with RNase).

## References

[1] Morales CP, Holt SE, Ouellette M, Kaur KJ, Yan Y (1999). Absence of cancer-associated changes in human fibroblasts immortalized with telomerase.. Nat Genet.

[2] Riethman H (2008). Human telomere structure and biology.. Annu Rev Genomics Hum Genet.

[3] Meyerson M (2000). Role of telomerase in normal and cancer cells.. J Clin Oncol.

[4] Stewart SA, Weinberg RA (2006). Telomeres: cancer to human aging.. Annu Rev Cell Dev Biol.

[5] Kishi S (2004). Functional aging and gradual senescence in zebrafish.. Ann N Y Acad Sci.

[6] Kishi S, Bayliss PE, Uchiyama J, Koshimizu E, Qi J (2008). The identification of zebrafish mutants showing alterations in senescence-associated biomarkers.. PLoS Genet.

[7] Kishi S, Uchiyama J, Baughman AM, Goto T, Lin MC (2003). The zebrafish as a vertebrate model of functional aging and very gradual senescence.. Exp Gerontol.

[8] McChesney PA, Elmore LW, Holt SE (2005). Vertebrate marine species as model systems for studying telomeres and telomerase.. Zebrafish.

[9] Nakatani Y, Kawakami A, Kudo A (2007). Cellular and molecular processes of regeneration, with special emphasis on fish fins.. Dev Growth Differ.

[10] Poss KD (2007). Getting to the heart of regeneration in zebrafish.. Semin Cell Dev Biol.

[11] Major RJ, Poss KD (2007). Zebrafish Heart Regeneration as a Model for Cardiac Tissue Repair.. Drug Discov Today Dis Models.

[12] Elmore LW, Norris MW, Sircar S, Bright AT, McChesney PA (2008). Upregulation of telomerase function during tissue regeneration.. Exp Biol Med (Maywood).

[13] Ulaner GA, Giudice LC (1997). Developmental regulation of telomerase activity in human fetal tissues during gestation.. Mol Hum Reprod.

[14] Lau BW, Wong AO, Tsao GS, So KF, Yip HK (2008). Molecular cloning and characterization of the zebrafish (Danio rerio) telomerase catalytic subunit (telomerase reverse transcriptase, TERT).. J Mol Neurosci.

[15] Hatakeyama H, Nakamura K, Izumiyama-Shimomura N, Ishii A, Tsuchida S (2008). The teleost Oryzias latipes shows telomere shortening with age despite considerable telomerase activity throughout life.. Mech Ageing Dev.

[16] Hartmann N, Reichwald K, Lechel A, Graf M, Kirschner J (2009). Telomeres shorten while Tert expression increases during ageing of the short-lived fish Nothobranchius furzeri.. Mech Ageing Dev.

[17] Kipling D, Ackford HE, Taylor BA, Cooke HJ (1991). Mouse minor satellite DNA genetically maps to the centromere and is physically linked to the proximal telomere.. Genomics.

[18] Kipling D, Cooke HJ (1992). Beginning or end? Telomere structure, genetics and biology.. Hum Mol Genet.

[19] Blasco MA, Lee HW, Hande MP, Samper E, Lansdorp PM (1997). Telomere shortening and tumor formation by mouse cells lacking telomerase RNA.. Cell.

[20] Modino S, Slijepcevic P (2002). Telomere shortening in mouse strains with constitutional chromosomal aberrations.. Int J Radiat Biol.

[21] Narducci ML, Grasselli A, Biasucci LM, Farsetti A, Mule A (2007). High telomerase activity in neutrophils from unstable coronary plaques.. J Am Coll Cardiol.

[22] Hathcock KS, Hemann MT, Opperman KK, Strong MA, Greider CW (2002). Haploinsufficiency of mTR results in defects in telomere elongation.. Proc Natl Acad Sci U S A.

[23] Mozdy AD, Cech TR (2006). Low abundance of telomerase in yeast: implications for telomerase haploinsufficiency.. RNA.

